# Kidney transplantation in infantile myofibromatosis and fibromuscular dysplasia: a case report

**DOI:** 10.1186/s13256-015-0756-8

**Published:** 2015-11-25

**Authors:** Julie Frezin, Fabio Fusaro, Raymond Reding, Nathalie Godefroid

**Affiliations:** 1 Department of Abdominal Surgery and Transplantation, Cliniques Universitaires Saint-Luc, Avenue Hippocrate, 10, 1200 Bruxelles, Belgium; 2 Pediatric Department, Cliniques Universitaires Saint-Luc, Avenue Hippocrate, 10, 1200 Bruxelles, Belgium

**Keywords:** Fibromuscular dysplasia, Infantile myofibromatosis, Kidney transplantation, Renovascular hypertension

## Abstract

**Introduction:**

We report what we believe to be the first case of a child affected by two rare vascular diseases complicated by kidney failure and successfully treated by kidney transplantation.

**Case presentation:**

A 3-year-old Caucasian girl with fibromuscular dysplasia and infantile myofibromatosis presented with arterial hypertension and renal failure. She received a deceased donor kidney transplantation distal to an iliac graft. The technical peculiarities of this transplantation are described, as well as her favorable long-term outcome.

**Conclusion:**

Kidney transplantation may be considered in a patient with vascular diseases and a history of iliac surgery.

## Introduction

Infantile myofibromatosis (IM) is a rare mesenchymal disorder characterized by the proliferation of tumors in the skin, muscles, bones and viscera. The generalized form involves multiple organs such as lung, kidney, heart, liver, adrenal glands, thyroid, gastrointestinal tract [1] and small vessels [2]. It has a poor prognosis with a mortality rate greater than 75 % [2, 3].

Fibromuscular dysplasia (FMD) is a fibrous non-atherosclerotic and non-inflammatory disease of the medium and small arteries [2]. It can be asymptomatic [4] or characterized by vascular dilatation or stenosis, and lead to arterial hypertension, aneurysms and thrombosis if left untreated [4, 5]. FMD is likely to be, at least in part, a genetic disorder [4]. It is the biggest cause of infantile renovascular hypertension, for which first treatment consists of antihypertensive medication and, in case of inefficacy, percutaneous transluminal angioplasty [6].

To the best of our knowledge, there are only two cases of associated IM and FMD reported in the literature, which is not sufficient to conclude a mutual origin [2, 3]. We report a case of renal failure in a child affected by both generalized IM and FMD, multiple venous thrombosis and renal failure. She was treated by kidney transplantation (KT) on a vessel-bearing prosthesis. The short outcome complications and treatment are discussed. The complexity and rarity of this case and the 5 years of follow up with good outcome make this report valuable.

## Case presentation

A 3-week-old Caucasian girl, born at 40 weeks after an uneventful pregnancy, was referred for a heart murmur and failure to thrive. A clinical examination showed increased blood pressure (170/110 mmHg, normal 60–80/30–50 mmHg) and a purple skin nodule on her right thigh. Echocardiography showed major left ventricular hypertrophy. A biopsy of the nodule was compatible with IM [2]. Radiological assessment, including abdominal magnetic resonance imaging (MRI), computed tomography of her chest, and a scintigraphic skeletal survey, showed diffuse muscular and visceral lesions. Chemotherapy with methotrexate and vinblastine was started and resulted in progressive regression of the lesions. Arterial hypertension was managed by propranolol and captopril. After 2 months, her creatinine clearance rate went down to 51.8 ml/min/m^2^ (Schwartz’s formula). A kidney color Doppler ultrasound showed a reduction in size of her right kidney with no diastolic flow in the artery. Mercaptoacetyltriglycine renal scintigraphy revealed a right-side relative renal function of only 30 %. Her glomerular filtration rate (GFR) was 68 mL/min/1.73 m^2^ (normal ±120 ml/min/1.73 m^2^).

**Fig. 1:**
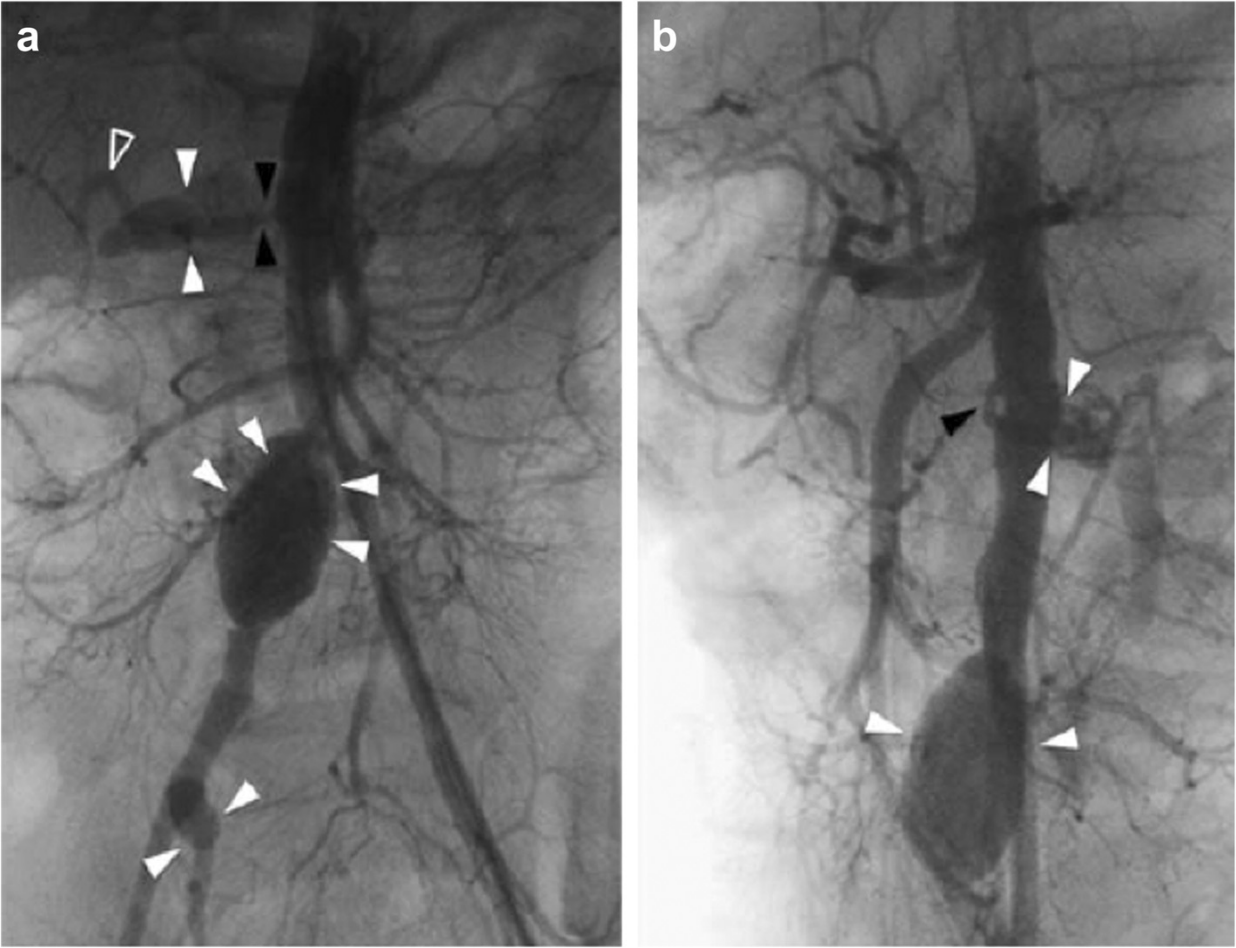
Renal angiography (**a** coronal, **b** sagittal) showing aneurismal dilatations of the right renal, the right common iliac and the right internal iliac arteries (*arrowheads*). The right renal artery shows a proximal stenosis (*black arrowheads*). The kidney is mainly perfused by a thin superior branch (*open arrowhead*)

**Fig. 2:**
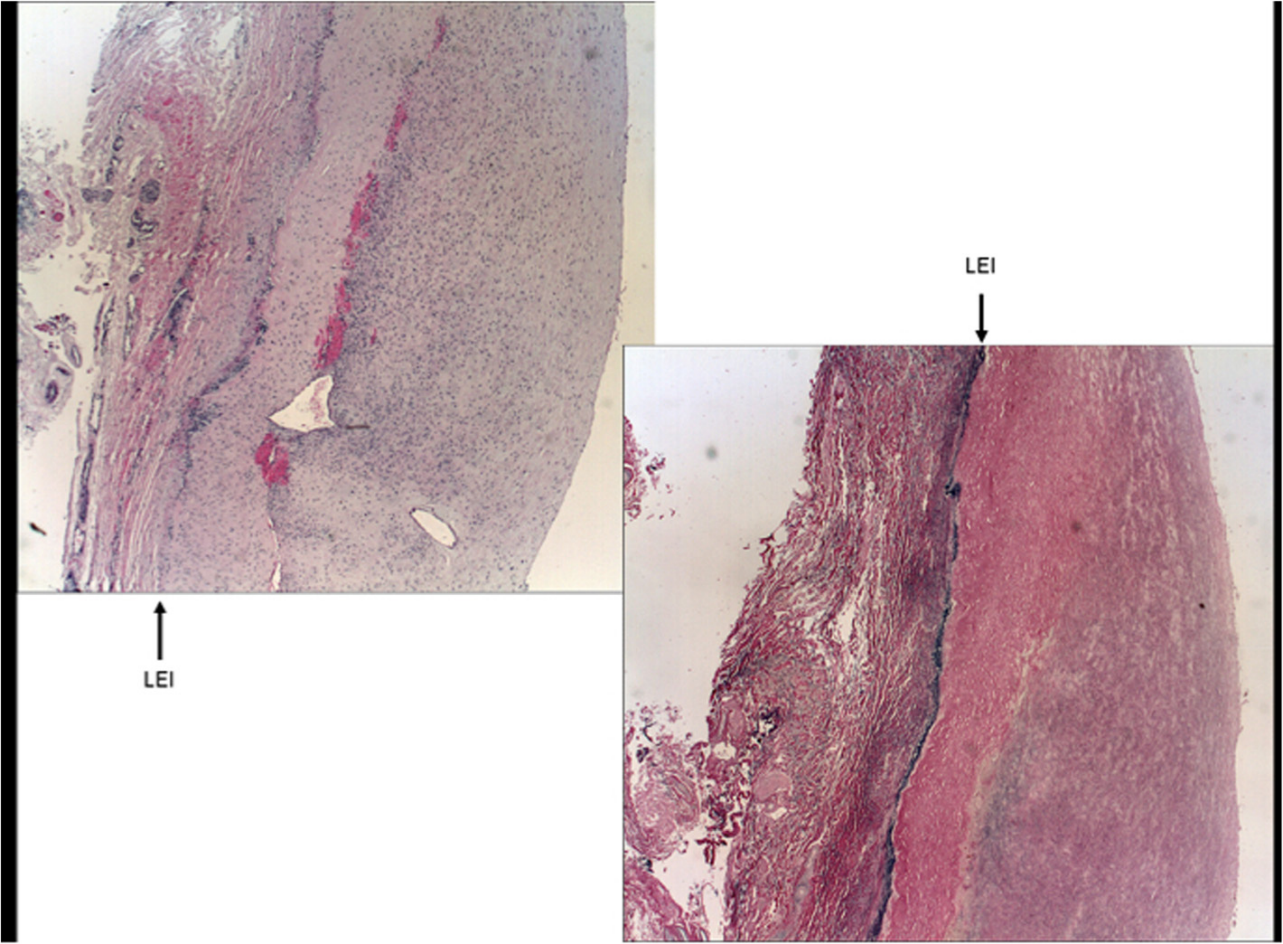
Left renal artery wall: thickened intima with relatively young conjunctive tissue. The internal elastic lamina is incomplete. The media is sclerotic with scarce elastic cells and the muscular cells are reduced in number. The adventitia is normal. This observation is compatible with mixed fibromuscular dysplasia, with both the intima and media being altered. *IEL*: Internal Elastica lamina

At 2 years of age, an angiography was performed because of the persistence of hypertension. This showed stenosis of her left renal artery with no perfusion of her left kidney, and two aneurisms involving her right renal artery and right common iliac artery (Fig. 1). Two Gore-tex grafts of 6 mm diameter each were interposed: the first one between her right adrenal gland artery and the superior polar branch of her right renal artery, and the second one half way along the length of her common iliac artery (Fig. 2). She received heparin with the following dosage regimen: 50 UI/kg/day on day 1, 220 UI/kg/day units on day 2, 280 UI/kg/day units on days 3 and 4, then stopped. However, on the first postoperative day, the renal bypass thrombosed and our patient developed irreversible kidney failure.

Hemodialysis was started with a catheter inserted in our patient’s right jugular vein thorough to the superior vena cava. After several weeks of hemodialysis, she developed an extensive thrombosis of her superior caval venous system. Peritoneal dialysis was started, which was complicated by chylous ascites. At 3 years of age (weight 11.7 kg), when her IM was considered to be in remission, she was registered on the waiting list for a KT.

The transplantation took place 2 months later, when an adult deceased donor became available (heart beating donor, weight 63 kg, height 173 cm, weight ratio 0.196). The kidney was connected to a perfusion machine for 4 hours until anastomosis. The kidney was transplanted on the right side using an extraperitoneal approach to preserve the peritoneum for dialysis. Because the ultrasound assessment showed no flow in the left iliac vein of the donor kidney, the left side was not considered for the transplantation. A vascular anastomosis was performed termino-lateral on the common iliac vein and common iliac artery below the prosthesis. Two running sutures of slowly absorbable 6.0 were used on each vessel. Her ureter was anastomosed to the bladder using the Lich-Gregoir procedure.

Our patient’s immunosuppressive protocol consisted of anti IL2R antibodies on days 0 and 4, methylprednisolone, mycophenolate mofetil, and tacrolimus (10 ng/ml of trough blood levels). Prophylaxis by subcutaneous administration of 200 UI/kg/day of low-molecular weight heparin was started. One month after the graft, the mycophenolate mofetil was switched to azathioprine. Subcutaneous nadroparin (low molecular weight heparin) was continued daily with diminishing doses.

The graft function normalized in 2 days (creatinine 0.69 mg/dl, GFR 71.3 ml/min/1.73 m^2^). Severe chylous ascites appeared on the ninth postoperative day with about 400 ml/day of chyle drained by the peritoneal dialysis catheter. Isotopic lymphography (Tc-99 m) and ultrasound confirmed a lymphatic leakage in her right iliac region and a lymphocele around the graft (65 × 48 × 92 mm) without vascular or ureteral compression. A conservative treatment was started using albumin and a non-fat diet, with stabilization of the chylous ascites after 8 weeks. Her blood pressure stayed high after the graft and was stabilized with amlodipine and enalapril. Our patient was then discharged with a stable clinical condition and good graft function (creatinine 0.39 mg/dl, GFR 126.2 ml/min/1.73 m^2^).

Three months after the transplantation, a polymerase chain reaction demonstrated a Epstein–Barr virus (EBV) count of over 200 000 copies/ml and her lymphocyte count had dropped to 240 cells/μL. To restore our patient’s immune response to EBV, the immunosuppressive regimen was decreased to methylprednisolone 2 mg/day and tacrolimus 1 mg twice a day (target blood concentration, 5 ng/ml), and azathioprine was stopped.

Four months after the transplantation, our patient developed chronic diarrhea. Blood tests showed severe hypoalbuminemia (1.9 g/dl). She was hospitalized because of the deterioration in her general condition, dyspnea and fever. A chest X-ray showed bilateral pleural effusion, which was drained. Her blood pressure was high and her anti-hypertensive treatment was therefore increased. Scintigraphy with transferrin In-111 revealed signs of exudative enteropathy. An upper gastrointestinal endoscopy was performed. A biopsy of her gastric mucosa confirmed a diagnosis of post-transplant lymphoproliferative disorder (monomorphic diffused large-B-cells lymphoma). Because our patient was not considered able to tolerate the COPADM standard chemotherapy protocol (cyclophosphamide, vincristine, doxorubicin, prednisone, methotrexate) and because of lymphocyte CD20 expression, treatment with three doses of anti-CD20 antibodies (rituximab) was chosen. Meanwhile, because of the persistence of her ascites and hypoalbuminemia, lymphatic MRI was done in another center. It showed a 70 × 55 × 78 mm lymphocele on the internal side of the graft, multiple thrombosis of the venous system with development of a collateral circulation, dilatation of her lymphatic system without visualization of a leak, and increased thickness of her intestinal wall. A gastric biopsy was done after the rituximab regimen and showed no residual sign of malignancy. She improved with parenteral nutrition and albumin supplementation, and was discharged home 6 months after transplantation.

At almost 5 years of follow-up (age 8), our patient is in good clinical condition (weight 25.6 kg, height 117 cm) and back at school. Her blood pressure is normal (106/64 mmHg) with atenolol, amlodipine and enalapril. An abdominal color Doppler ultrasound showed a normal aspect of the graft, with lymphocele and ascites resolution. Her creatinine level was 0.40 mg/dl with a calculated GFR of 139.7 ml/min/1.73 m^2^. Fraxiparine continues at a dosage of 60 UI/kg/day.

## Discussion

Some studies have tried to assess the natural evolution of FMD, showing worsening stenosis in 33 % but no cases of complete arterial obstruction. However, methodological problems have been found in those studies, with a risk of progression that may have been overestimated [4, 5]. When affecting a lot of vessels, FMD can be rapidly progressive, particularly in young children [5]. Renal autotransplantation is an accepted surgical treatment for FMD with involvement of renal vessels, providing good long-term results with a return to normal blood pressure [3, 7, 8].

Retrospectively, the surgical decision to repair the right renal artery in our patient may be discussed because it made the KT more difficult, but at that time, the main objective was to save the right kidney. When faced with a case of diffuse vascular disease, a vascular preservation strategy is paramount. Moreover, renal artery reconstruction using an autologous graft is known to have better results than when using a prosthetic, because of a major risk of thrombosis in a prosthetic graft because they do not grow with the child [9, 10]. It was for this reason that the vascular surgeon in our case chose a graft with a larger diameter than that of the vessel diameter. Indeed, Barral *et al*. state the need to wait to the end of growth before repairing vessels if possible. If not, an allograft is better than a prosthesis [10]. These authors advise that the external anastomosis of the graft be reinforced and for the graft to be over-long to compensate for the growth of the child. However, few reports concerning the use of vascular prosthesis in children have been published so it was difficult to predict the evolution of our patient based on the literature. Also, the transplantation was done on a vessel previously repaired with a prosthesis. We found no relevant publications on that subject for pediatric cases but there are reports citing good results from KTs simultaneously performed with vascular repair in severe cases of adult arteriosclerosis [11, 12].

Diffuse venous thrombosis and FMD constitute a rare indication for KT [13]. Our choice to proceed with transplantation despite a complicated and mostly thrombosed venous network was greatly motivated by the normal psychomotor development of the child. Our patient lost both kidneys because both renal arteries were affected by the FMD and she developed an aneurysm on the common iliac artery, which is unusual in FMD [14]. The graft artery was anastomosed on the common iliac artery below the reconstructed vascular segment. We decided to do so because of the normal macroscopic aspect of the vessel and a normal frozen section. Two years after transplantation, the graft and iliac vessels were normal on a color Doppler ultrasound. Fraxiparine prophylaxis was administered after transplantation to prevent venous thrombosis of the graft. Our patient presented other risk factors for KT, such as weight under 15 kg and pretransplantation use of hemodialysis. Young children tend to have more delayed graft function and graft thrombosis but less rejection [15, 16]. The graft function in our patient restored without delay and neither graft thrombosis nor rejection was seen.

There is only one other reported case of successful KT in a patient with FMD in the medical literature, which showed good kidney function after 8 years of follow-up and blood pressure normalized with only enalapril [6]. However, that transplantation was performed in a 6-year-old patient, without a history of infantile myofibromatosis, chemotherapy or peritoneal dialysis and without anastomosis on a repaired vessel. Also, bilateral nephrectomy was performed before KT to control hypertension. Nevertheless, the good results of that case support us in our choice for KT in our patient.

## Conclusion

We present the complex case of a 3-year-old girl with IM and FMD. Those conditions led to arterial hypertension, superior vena cava syndrome, renal and iliac aneurysms, and finally renal failure. A KT was performed with a deceased adult donor kidney graft on a repaired iliac artery bearing a prosthesis and with a thrombosed inferior vena cava. The short-term complications and their treatments are discussed. After 5 years of follow-up, our patient has a normal renal function. We consider KT to be a therapeutic option in patients with renal failure caused by these rare vascular diseases. Long-term use of efficient anti-coagulant therapy is required.

## Consent

Written informed consent was obtained from the parents of the child for publication of this case report and accompanying images. A copy of the written consent is available for review by the Editor-in-Chief of this journal.

## Abbreviations

EBV: Epstein–Barr virus
FMD: fibromuscular dysplasia
GFR: glomerular filtration rate
IM: infantile myofibromatosis
KT: kidney transplantation
MRI: magnetic resonance imaging
PCR: polymerase chain reaction
